# Empowering nurse leaders: readiness for AI integration and the perceived benefits of predictive analytics

**DOI:** 10.1186/s12912-024-02653-x

**Published:** 2025-01-16

**Authors:** Mohamed Hashem Kotp, Hossam Ali Ismail, Hassan Ahmed Awad Basyouny, Mohamed Ahmed Aly, Abdelaziz Hendy, Abdulqadir J. Nashwan, Ahmed Hendy, Aliaa Ezz Eldin Abd Elmoaty

**Affiliations:** 1 Nursing Administration, Faculty of Nursing, Helwan University, Cairo, Egypt; 2 Nursing Administration, Faculty of Nursing, Helwan University, Cairo, Egypt; 3 Nursing Administration, Faculty of Nursing, Helwan University, Cairo, Egypt; 4 Nursing Administration, Faculty of Nursing, Helwan University, Cairo, Egypt; 5 Prince Sattam Bin Abdulaziz University, Cairo, Wadi Addawasir, Saudi Arabia; 6 Pediatric Nursing, Faculty Nursing, Ain Shams University, Cairo, Egypt; 7 Nursing & Midwifery Research Department (NMRD), Hamad Medical Corporation, Doha, Qatar; 8 Department of Public Health, College of Health Sciences, QU Health, Qatar University, Doha, Qatar; 9 Department of Computational Mathematics and Computer Science, Institute of Natural Sciences and Mathematics, Ural Federal University, Yekaterinburg, 620002 Russian Federation; 10 Department of Mechanics and Mathematics, Western Caspian University, Baku, 1001 Azerbaijan; 11 Nursing Administration, Faculty of Nursing, Helwan University, Cairo, Egypt

**Keywords:** Artificial intelligence, Predictive analytics, Nursing leadership, Decision support systems, Patient care management, Nursing informatics

## Abstract

**Introduction:**

Artificial Intelligence (AI) is increasingly being integrated into healthcare, particularly through predictive analytics that can enhance patient care and operational efficiency. Nursing leaders play a crucial role in the successful adoption of these technologies.

**Aim:**

This study aims to assess the readiness of nursing leaders for AI integration and evaluate their perceptions of the benefits of AI-driven predictive analytics in healthcare.

**Methods:**

A descriptive cross-sectional study was conducted among 187 nurse leaders across nine private hospitals in Cairo. The sample was selected using a combination of simple random sampling and non-probability convenience sampling methods to ensure a diverse representation of nursing leadership. Data collection took place from March to May 2024, utilizing a structured questionnaire specifically designed to assess nurse leaders’ readiness for AI integration and their perceptions of AI-driven predictive analytics The data were analyzed using IBM SPSS Statistics, version 26.0. Exploratory Factor Analysis (EFA) was employed to identify underlying factors related to readiness and perceived benefits. Confirmatory Factor Analysis (CFA) was subsequently performed to validate the factor structure. Multiple linear regression analysis was conducted to identify significant predictors of AI readiness and perceived benefits.

**Results:**

The study revealed that over one-third of nurse leaders exhibited high readiness for AI integration. Significant predictors of readiness included age, educational attainment, and employment status. Positive correlations were found between readiness and perceived benefits of AI, particularly in areas such as care planning and decision-making.

**Conclusion:**

The findings suggest that nursing leaders are generally prepared to integrate AI into their workflows, especially those with advanced education and experience. However, further training and policy development are necessary to fully realize the benefits of AI in nursing practice.

**Supplementary Information:**

The online version contains supplementary material available at 10.1186/s12912-024-02653-x.

## Introduction

In the rapidly changing and advancing field of healthcare, Artificial Intelligence (AI) has emerged as a transformative force, particularly in the field of nursing. The integration of AI-driven predictive analytics in nursing is revolutionizing patient care by enabling anticipatory and personalized healthcare solutions [1]. Predictive analytics leverages massive amounts of data to forecast future events and trends, providing critical insights that can enhance decision-making processes in healthcare [2].

The implementation of AI in nursing is not without challenges. There are concerns regarding data privacy, the accuracy of AI predictions, and the potential for technology to depersonalize patient care [3]. However, when implemented with careful consideration of these factors, AI can significantly enhance the efficiency and effectiveness of nursing care. For instance, AI can streamline administrative tasks, allowing nurses to focus more on direct patient care [4].

Leadership plays a crucial role in the successful integration of AI in nursing [5]. Effective leaders can foster a culture of innovation and continuous learning, ensuring that nursing staff are well-equipped to utilize AI tools. Approaches that emphasize leadership collaboration, ongoing education, and ethical considerations are essential for navigating the complexities associated with AI adoption [6]​.

One of the primary benefits of predictive analytics in nursing is its potential to improve patient outcomes through anticipatory care [7]​. By analyzing patient data in real-time, AI can identify subtle changes in a patient’s condition that may indicate the onset of a serious issue. This allows healthcare providers to intervene early, potentially saving lives and reducing healthcare costs​ [8]. Moreover, predictive analytics can enhance patient centered care by tailoring care plans to individual patients based on their unique genetic makeup, lifestyle, and medical history [9].

At the leadership level, integrating AI in nursing also significantly improves operational efficiency [10]. Predictive analytics can optimize staffing levels, reduce hospital wastages, and improve the management of healthcare resources. These efficiencies can lead to cost savings and better allocation of resources, ultimately benefiting both healthcare providers and patients [11].

Despite the potential benefits, integrating AI in nursing needs addressing a few challenges. These include the need for robust data governance frameworks, ensuring the interoperability of AI systems with existing healthcare technologies, and providing adequate training for nursing staff [12]. Leaders must also consider the ethical implications of AI, such as the potential for bias in AI algorithms and the need to maintain patient trust and confidentiality [13, 14].

Furthermore, the success of AI-driven predictive analytics in nursing depends on the quality and completeness of the data used. Inaccurate or incomplete data can lead to misleading predictions, which can have serious consequences on patient and operational outcomes [15, 16]. Therefore, healthcare organizations must invest in data quality initiatives and ensure that data collected is accurate, comprehensive, and up-to-date​ [17].

In addition to improving patient care and management outcomes, AI can enhance the professional development of nurses by providing them with advanced tools and resources for learning [5, 18]. AI-powered educational platforms can offer personalized learning experiences, helping nurses to stay updated with the latest medical knowledge and best practices [19]. By analyzing large datasets, researchers can gain new insights into disease patterns, treatment outcomes, and healthcare delivery models. These insights can drive the development of new interventions and improve the overall quality of care​ [20].

Using AI in nursing can enhance the ability of healthcare providers to deliver personalized care. By leveraging data on individual patients, AI can tailor treatment plans to meet the specific needs of each patient. This personalized approach has been shown to improve the effectiveness of treatments and increase patient satisfaction. As healthcare moves towards more patient-centered models of care, the role of AI in enabling these models will become increasingly important [21, 22].

By enabling anticipatory care, AI can dramatically improve patient outcomes while reducing healthcare costs. This proactive approach to healthcare allows for early intervention, preventing the progression of diseases and minimizing the need for costly treatments and hospitalizations. This not only benefits patients but also alleviates the burden on healthcare systems, making care more sustainable and efficient [23]​.

Understanding nursing leaders’ perceptions of AI-driven predictive analytics is critical for the successful implementation and integration of these technologies in healthcare settings. Positive perceptions among leaders facilitate smoother transitions, increase staff engagement, and ensure the full realization of AI’s potential benefits [24, 25]. Leaders who support AI can drive policy development and strategic planning, advocating for necessary investments and developing ethical use protocols [26, 27]. Understanding nursing leaders’ perceptions is vital for improving patient outcomes, enhancing operational efficiency, and supporting continuous professional development in nursing [28, 29].​.

## Methods

### Research aim

To assess the readiness of nursing leaders for the integration of AI-driven predictive analytics in healthcare settings and to evaluate their perceived benefits of AI in enhancing patient care, decision-making, and clinical outcomes.

#### Research questions

1. What is the current level of readiness among nursing leaders for integrating AI-driven predictive analytics into their workflows?
2. How do nursing leaders perceive the benefits of AI-driven predictive analytics in improving patient care and clinical decision-making?
3. What are the key factors that influence nursing leaders’ readiness for AI adoption in healthcare?
4. How does the perceived readiness of nursing leaders correlate with their perceived benefits of AI-driven predictive analytics?

#### Research design, sample, and sampling

**Fig. 1:**
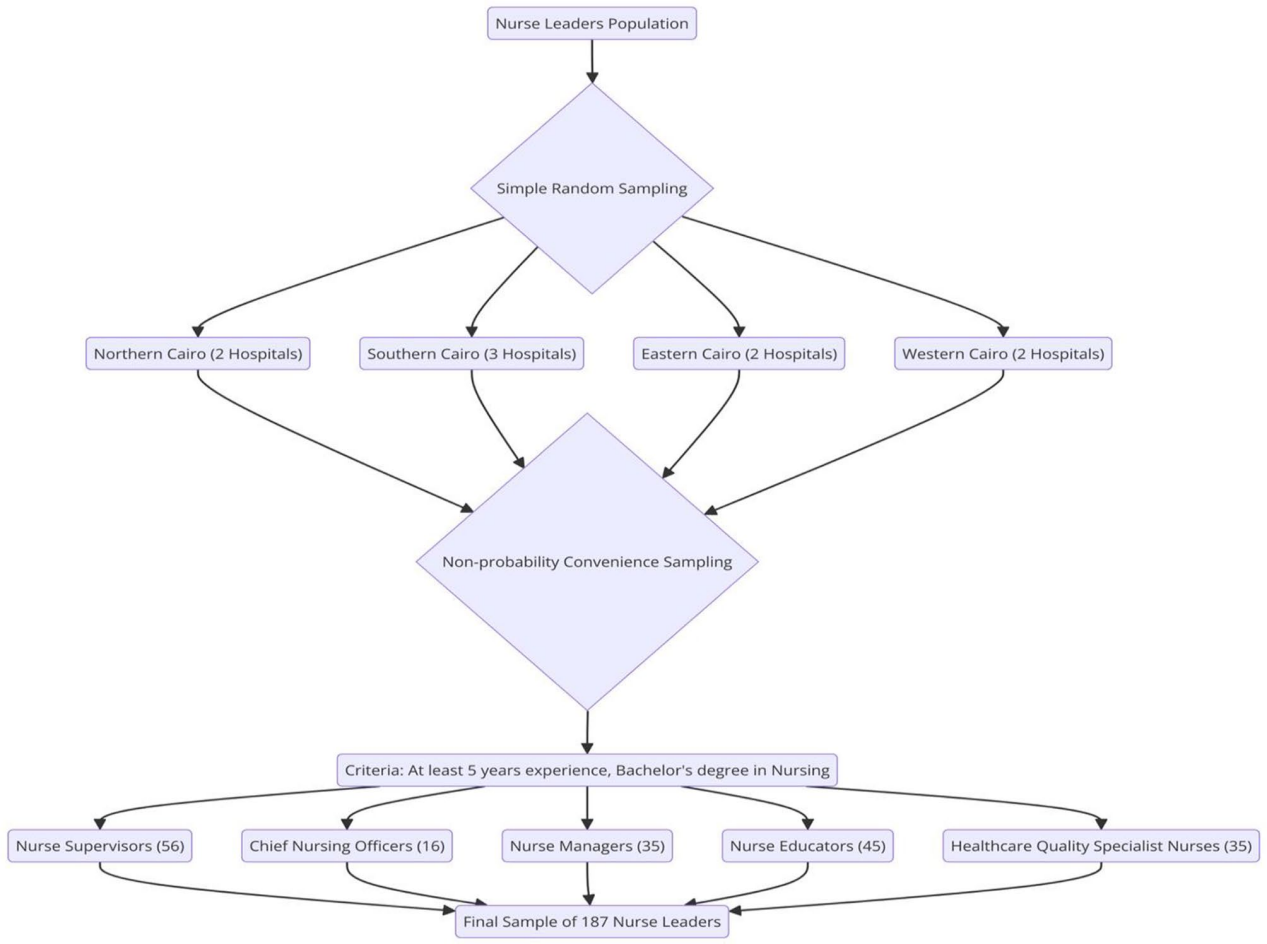
Sampling Process for Selecting Nurse Leaders

A descriptive cross-sectional research design was conducted on 187 nurse leaders from 9 private hospitals in Cairo. The hospitals were selected through a multistage sampling process. In the first stage, hospitals were chosen using a simple random sampling technique from the four main divisions of Cairo Governorate: Northern Cairo (2 hospitals), Southern Cairo (3 hospitals), Eastern Cairo (2 hospitals), and Western Cairo (2 hospitals). Hospitals with fewer than 70 beds were excluded due to the limited number of registered nurses. In the second stage, nurse leaders were selected using a non-probability convenience sampling method, focusing on individuals in roles such as Nurse Supervisors (56), Chief Nursing Officers (16), Nurse Managers (35), Nurse Educators (45), and Healthcare Quality Specialist Nurses (35). Eligibility criteria for the study required nurse leaders to have at least a bachelor’s degree in nursing and a minimum of five years of nursing experience, as mentioned in Fig. 1. The sample size was estimated using Cochran’s formula (Cochran, 1977) [30].:

The sample size was calculated using Cochran’s formula for sample size determination, which is expressed as follows:


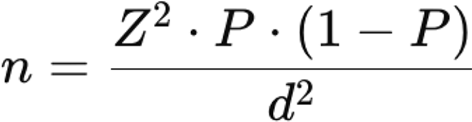


**Where**:

- *n*: Required sample size.
- *Z*: Z-value corresponding to the desired confidence level (1.96 for 95%).
- *P*: Estimated proportion (assumed to be 0.5 for maximum variability).
- *d*: Margin of error (0.05).

The target population for the study was 318 nurse leaders. Based on Cochran’s formula, the initial estimated sample size was 175 participants. To account for potential non-response or incomplete questionnaires, an additional 12 participants were added (175 + 12 = 187). Subsequently, to ensure a robust response rate, the total number of invitations sent out was increased to 200. This adjustment aimed to achieve the desired sample size of 187 completed responses, resulting in a response rate of 93.5%.

## Data collection procedures

The recruitment of nurse leaders for this study was conducted through professional and neutral channels to ensure voluntary participation and minimize potential bias or coercion. Eligible nurse leaders were identified through their respective hospital administrations, which provided a list of individuals meeting the inclusion criteria. A standardized email invitation was sent to potential participants by the research team, outlining the study’s purpose, eligibility criteria, assurances of confidentiality and anonymity, and a direct link to the study questionnaire. Email was the primary method of communication; however, in cases where email was unavailable, invitations were shared through secure internal messaging systems used by the hospitals, such as WhatsApp or internal communication portals. Participants were explicitly informed that their involvement in the study was entirely voluntary and that their decision to participate or withdraw would not impact their professional roles or relationships with colleagues or supervisors. To maximize response rates, a single reminder was sent via the same communication channel initially used, ensuring no direct personal follow-ups were conducted to avoid any perception of coercion. Access to the online survey was granted only after participants provided electronic informed consent through the questionnaire platform.

Data collection was conducted from March to May 2024. To facilitate convenient scheduling, the researchers obtained lists of hospitals and nurse leaders along with their work schedules. After a screening process to confirm eligibility, nurse leaders were invited to participate by completing a questionnaire. Approval for the study was secured prior to data collection. The questionnaire was personally distributed to nurse leaders who met the inclusion criteria while they were on duty. The purpose of the study was clearly explained, and the voluntary and confidential nature of participation was emphasized on the first page of the questionnaire. Completion of the survey indicated the participants’ consent to take part in the research. The questionnaire required approximately 20–25 min to complete.

## Measurements

### Exploratory factor analysis (EFA)

Bartlett’s test of sphericity was performed to determine the adequacy of the data for factor analysis, yielding a significant result (*p* < 0.001). Additionally, the Kaiser-Meyer-Olkin (KMO) measure of sampling adequacy was calculated and surpassed the minimum acceptable value of 0.7, confirming the suitability of the dataset for factor analysis. A principal component analysis (PCA) with varimax rotation was conducted on the data from the Nurse Leaders’ Readiness for AI Integration Survey (20 items) and the Perceived Benefits Survey (10 items), as mentioned in Tables 3 and 4.

The exploratory factor analysis (EFA) of the Nurse Leaders’ Readiness for AI Integration Survey identified three common factors, explaining a significant portion of the total variance. All 20 items loaded significantly onto these three factors, with factor loadings ranging from 0.760 to 0.842.

-Factor 1 was labeled “Leadership Initiatives,” capturing items related to proactive leadership support, strategic AI goals, and alignment of AI initiatives with the organization’s mission.
-Factor 2 was labeled “Staff Engagement,” reflecting staff openness, enthusiasm, and collaboration regarding AI technologies, as well as the perceived benefits of AI in improving patient outcomes.
-Factor 3 was termed “Technical Readiness,” focusing on the necessary technical infrastructure, system support for AI integration, and data security.

The exploratory factor analysis (EFA) of the Perceived Benefits of AI-Driven Predictive Analytics Survey identified two common factors, explaining a significant portion of the total variance. All 10 items loaded significantly onto these two factors, with factor loadings ranging from 0.756 to 0.836.

- Factor 1 was labeled “Patient Care & Outcomes,” which encompassed items related to the enhancement of patient care through real-time predictive insights, anticipation of patient needs, reduction of complications, optimization of treatment plans, and personalization of care based on predictive data.
- Factor 2 was labeled “Decision-Making Support,” capturing items focused on improving the decision-making process by providing data-driven recommendations, reducing uncertainty, integrating with existing decision-support systems, and enabling faster and more accurate decisions.

Together, these factors reflect the multifaceted benefits of AI-driven predictive analytics in enhancing patient care and supporting effective decision-making in nursing leadership.

### Confirmatory Factor Analysis (CFA)

Confirmatory Factor Analysis (CFA) was performed on a sample of 187 nurse leaders to validate the structure of two surveys: the Nurse Leaders’ Readiness for AI Integration Survey (three-factor model) and the Perceived Benefits of AI-Driven Predictive Analytics Survey (two-factor model). The results indicated that the three-factor model for the Nurse Leaders’ Readiness survey had a good fit with the data, as evidenced by the following fit indices: χ²/df = 2.945, CFI = 0.950, GFI = 0.920, AGFI = 0.892, RMSEA = 0.069, and RMR = 0.054. These indices demonstrate a robust model fit, with values aligning closely with the ideal standards for CFI, GFI, and RMSEA.

Similarly, the two-factor model for the Perceived Benefits Survey showed an acceptable fit, with fit indices of χ²/df = 2.732, CFI = 0.953, GFI = 0.928, AGFI = 0.887, RMSEA = 0.067, and RMR = 0.050. The high CFI and GFI values, coupled with low RMSEA and RMR, confirm the validity of this model in evaluating perceived benefits, particularly in areas related to patient care, outcomes, and decision-making support. Overall, the CFA results endorse the reliability and validity of both models, indicating they effectively measure the intended constructs, see more in Table 1.

**Table 1:** Confirmatory factor analysis (*N* = 187)

Fit Indices	Nurse Leaders’ Readiness for AI Integration (Three-Factor Model)	Perceived Benefits (Two-Factor Model)
χ²/df	2.945	2.732
Comparative Fit Index (CFI)	0.950	0.953
Goodness of Fit Index (GFI)	0.920	0.928
Adjusted GFI (AGFI)	0.892	0.887
RMSEA	0.069	0.067
RMR	0.054	0.050

## Reliability analysis

The internal consistency of the scales was assessed using Cronbach’s alpha, with the Nurse Leaders’ Readiness for AI Integration Survey showing an overall Cronbach’s alpha of 0.90, indicating high internal consistency. Within this survey, Factor 1 (Leadership Initiatives) had a Cronbach’s alpha of 0.91, Factor 2 (Staff Engagement) had an alpha of 0.89, and Factor 3 (Technical Readiness) exhibited an alpha of 0.88. The Perceived Benefits of AI-Driven Predictive Analytics Survey demonstrated a Cronbach’s alpha of 0.89, confirming strong internal reliability, with Factor 1 (Patient Care & Outcomes) showing a Cronbach’s alpha of 0.88 and Factor 2 (Decision-Making Support) having an alpha of 0.87. To evaluate test-retest reliability, a random subset of 30 nurse leaders completed the surveys again two weeks after the initial administration, with the Nurse Leaders’ Readiness for AI Integration Survey achieving a Spearman correlation coefficient of 0.854 and the Perceived Benefits of AI-Driven Predictive Analytics Survey achieving a Spearman correlation of 0.834, both surpassing the 0.7 threshold for reliability. These results confirm that both surveys are reliable tools for assessing nursing leaders’ readiness for AI integration and their perceptions of AI benefits over time, see more in Table 2.

**Table 2:** Reliability analysis (internal consistency and test-retest reliability)

Scale	Cronbach’s Alpha	Test-Retest Reliability (Spearman)
Nurse Leaders’ Readiness for AI Integration (Overall)	0.90	0.854
- Leadership Initiatives (Factor 1)	0.91	
- Staff Engagement (Factor 2)	0.89	
- Technical Readiness (Factor 3)	0.88	
Perceived Benefits of AI-Driven Predictive Analytics	0.89	0.834
- Patient Care & Outcomes (Factor 1)	0.88	
- Decision-Making Support (Factor 2)	0.87	

**Table 3:** Exploratory Factor Analysis (EFA) results for nursing leaders’ readiness for AI integration (*N* = 187)

Item Description	Factor 1: Leadership Initiatives	Factor 2: Staff Engagement	Factor 3: Technical Readiness	Factor Loadings
Leadership is proactive in supporting AI implementation	0.781	-	-	0.781
Leadership encourages innovation related to AI	0.794	-	-	0.794
Leadership sets clear AI goals for the organization	0.816	-	-	0.816
AI implementation is a strategic priority in our organization	0.760	-	-	0.760
Leadership communicates AI benefits to staff	0.783	-	-	0.783
Staff members are open to learning about AI	-	0.824	-	0.824
Staff are enthusiastic about new AI technologies	-	0.793	-	0.793
Staff are confident in using AI in day-to-day tasks	-	0.813	-	0.813
Staff collaborate effectively when implementing AI solutions	-	0.799	-	0.799
Staff are regularly trained in AI technologies	-	0.828	-	0.828
We have the necessary technical infrastructure for AI	-	-	0.842	0.842
Our systems are equipped to support AI integration	-	-	0.825	0.825
AI technology is effectively integrated into our existing workflows	-	-	0.806	0.806
We update our AI technology regularly	-	-	0.790	0.790
We have access to the resources needed for successful AI implementation	-	-	0.812	0.812
Our AI systems are secure and protect patient data	-	-	0.804	0.804
We use data-driven insights from AI to inform decision-making	-	-	0.796	0.796
Leadership encourages AI training and development for staff	0.772	-	-	0.772
AI helps us improve patient outcomes	-	0.784	-	0.784
AI initiatives are aligned with our organization’s overall mission	0.768	-	-	0.768

**Table 4:** Exploratory Factor Analysis (EFA) results for Perceived benefits of AI-Driven predictive analytics (*N* = 187)

Item Description	Factor 1: Patient Care & Outcomes	Factor 2: Decision-Making Support	Factor Loadings
AI enhances patient care by providing real-time predictive insights	0.823	-	0.823
AI helps in anticipating patient needs and improving outcomes	0.791	-	0.791
AI-driven analytics contributes to reducing patient complications	0.812	-	0.812
AI assists in optimizing patient treatment plans	0.801	-	0.801
AI helps identify potential risks before they become critical	0.774	-	0.774
AI enhances the decision-making process by providing data-driven recommendations	-	0.836	0.836
AI reduces uncertainty in decision-making during patient care planning	-	0.793	0.793
AI allows for faster and more accurate decision-making	-	0.821	0.821
AI tools integrate well with existing decision-support systems	-	0.809	0.809
AI helps to personalize patient care based on predictive data	0.756	-	0.756

## Tools

The survey instrument used in this study was an online questionnaire developed by the researchers to assess nursing leaders’ preparedness for integrating AI-driven predictive analytics into their workflows. The development process involved a comprehensive review of relevant studies, guidelines, and existing frameworks in the field of AI integration in healthcare. The questionnaire was deployed using a secure online platform (Google Forms), ensuring accessibility and convenience for participants. The survey link was shared with participants via email and other secure communication channels Tables 3 and 4.

### Socio demographic characteristics

A demographic questionnaire assessed the participants’ characteristics including gender, age, highest nursing degree, and employment status.

### Nursing leaders’ readiness for AI-Driven predictive analytics

The researchers developed a questionnaire to assess nursing leaders’ preparedness for integrating AI-driven predictive analytics into their workflows after reviewing many studies (26, 27, 28, 29). This questionnaire consisted of 20 items and was answered using a 4-point Likert scale: 1 = strongly disagree, 2 = disagree, 3 = agree, 4 = strongly agree, see full items in attached file. Higher scores indicated a better-perceived readiness. The tool underwent content validity testing with five nursing experts. The total possible score was 80, with respondents scoring above 60 considered to have a high level of readiness, scores between 40 and 60 indicating a medium level of readiness, and scores below 40 considered as low readiness.

### Perceived benefits Survey of AI-Driven predictive analytics

This survey was developed by researchers after reviewing many studies (25, 27, 28, 30) to assess nursing leaders’ perceived benefits of AI-driven predictive analytics in enhancing patient care planning, decision-making, and outcomes. The survey consisted of 10 items, each answered on a 5-point Likert scale: 1 = strongly disagree, 2 = disagree, 3 = neutral, 4 = agree, and 5 = strongly agree, see full items in attached file. The total possible score was 50, with higher scores indicating a greater perceived benefit of AI.

The questionnaire’s development followed a structured process to ensure validity and reliability. Before deployment, the instrument underwent content validity testing by a panel of five nursing and AI experts. These experts evaluated the relevance, clarity, and comprehensiveness of each item to ensure it appropriately addressed the study objectives. The feedback provided by the experts was incorporated into the final questionnaire.

To ensure face validity and refine the questionnaire for clarity and usability, a pilot study was conducted with 20 nurse leaders who were not part of the final sample. This pilot testing helped identify ambiguities, adjust the phrasing of certain items, and ensure that the online format was user-friendly. The pilot data were not included in the final analysis.

### Data analysis

The statistical analysis for this study was conducted using IBM SPSS Statistics, version 26.0, focusing on both univariate and multivariate techniques to examine the relationships between sociodemographic characteristics, readiness for AI integration, and perceived benefits of AI among nursing leadership. Exploratory Factor Analysis (EFA): To determine the suitability of the dataset for factor analysis, Bartlett’s test of sphericity yielded a significant result (*p* < 0.001), and the Kaiser-Meyer-Olkin (KMO) measure of sampling adequacy surpassed 0.7. Confirmatory Factor Analysis (CFA): CFA was performed on both surveys to validate the identified factors, with fit indices indicating a robust model fit. Cronbach’s alpha was used to assess internal consistency. Both t-tests and ANOVA (Analysis of Variance) were employed to compare the means of different groups and assess the significance of differences based on sociodemographic characteristics. Two multiple linear regression analyses were conducted to identify significant predictors of readiness for AI integration and perceived benefits of AI. A significance level of *p* < 0.05 was deemed statistically significant.

## Results

**Table 5:** Sociodemographic characteristics of the studied subjects (*N* = 187)

Sociodemographic Characteristics	*N*	(%)	Readiness For AI Integration (Mean ± SD)	*P*	Perceived Benefits of AI (Mean ± SD)	*P*
**Gender**						
Male	136	72.7	64.21 ± 10.42	0.214	78.79 ± 9.19	0.101
Female	51	27.3	66.17 ± 12.88		81.27 ± 8.88	
**Age**						
Less than 35 years	35	18.7	62.00 ± 8.13	0.048*	76.85 ± 10.51	0.022*
35–40 years	108	57.8	67.13 ± 10.93		82.27 ± 9.85	
> 40 years	44	23.5	70.55 ± 11.76		85.04 ± 8.46	
**Highest Nursing Degree Earned**						
Bachelor of Science in Nursing (BSN)	14	7.5	59.00 ± 9.85	0.046	77.55 ± 10.90	0.0198*
Master of Science in Nursing (MSN)	124	66.3	68.71 ± 10.90		83.21 ± 9.42	
Nursing PhD.	49	26.2	71.75 ± 11.87		84.12 ± 9.98	
**Current Employment Status**						
Nurse supervisor	56	29.9	64.15 ± 10.43	0.029*	79.00 ± 9.55	0.031*
Chief Nursing Officer	16	8.6	66.00 ± 10.66		80.75 ± 9.30	
Nurse manager	35	18.7	68.25 ± 9.60		81.65 ± 8.58	
Nurse Educator	45	24.1	69.00 ± 10.96		82.83 ± 9.98	
Healthcare quality specialist nurse	35	18.7	70.00 ± 10.43		83.60 ± 10.05	

The Table (5) provides an overview of the sociodemographic characteristics of the 187 studied subjects and their association with readiness for AI integration and perceived benefits of AI. The data indicates that gender does not significantly impact either readiness or perceived benefits, as evidenced by the similar mean scores and non-significant p-values. However, age plays a significant role, with older individuals (over 40 years) showing higher readiness for AI integration (*p* = 0.048) and greater perceived benefits (*p* = 0.022). Similarly, higher educational attainment, particularly holding a PhD, is associated with greater readiness and perceived benefits of AI, with significant p-values (*p* = 0.046 and *p* = 0.0198, respectively). Employment status also significantly influences these factors, with healthcare quality specialist nurses showing the highest readiness and perceived benefits (*p* = 0.029 and *p* = 0.031, respectively). These findings suggest that age, educational level, and current employment status are important determinants of how nursing leadership perceives and prepares for AI integration in their professional roles.

**Table 6:** Readiness for AI integration Among Nursing Leadership (*N* = 187)

Readiness Level	*N*	(%)	Mean ± SD
Low	55	29.2	65.05 ± 11.08
Moderate	63	34.0	
High	69	36.8	

The Table 6. provides insights into the readiness for AI integration among nursing leadership, based on a sample of 187 respondents. The data is categorized into three levels of readiness: low, moderate, and high. Approximately 29.2% of the respondents fall into the low readiness category, with a mean score of 65.05 ± 11.08. A slightly higher percentage, 34.0%, are classified as having moderate readiness, while the largest group, 36.8%, is considered highly ready for AI integration.

The table presents descriptive statistics for the perceived benefits of AI-driven predictive analytics among a sample size of 187 respondents. The results indicate a high perception of benefits in areas such as “Improved Care Planning” (Mean = 4.06, SD = 0.42) and “Enhanced Decision-making” (Mean = 4.03, SD = 0.45), with over 74.9% and 85.0% of participants, respectively, acknowledging these as significant advantages. Other notable benefits include “Better Patient Outcomes” and “Personalized Patient Care,” with mean scores above 4.0, reflecting strong agreement among respondents. However, benefits such as “Reduced Length of Stay” and “Supports Population Health Management” were perceived less favorably, indicated by lower mean scores of 3.63 and 3.67, respectively, see more Table 7.

**Table 7:** Perceived benefits of AI-Driven predictive analytics among nursing Leadership (*N* = 187)

Perceived Benefits	*N*	(%)	Mean ± SD
Improved Care Planning	140	74.9	4.06 ± 0.42
Enhanced Decision-making	159	85.0	4.03 ± 0.45
Better Patient Outcomes	147	78.6	4.05 ± 0.44
Cost Savings	94	50.3	3.84 ± 0.47
Enhanced Workflow Efficiency	117	62.5	3.86 ± 0.51
Reduced Length of Stay	84	44.9	3.63 ± 0.36
Predicting Adverse Events	114	61.0	3.78 ± 0.37
Personalized Patient Care	130	69.5	3.91 ± 0.45
Facilitates Resource Allocation	104	55.6	3.83 ± 0.44
Supports Population Health Management	95	50.8	3.67 ± 0.41
**Total**	187	100	39.66 ± 4.32

**Fig. 2:**
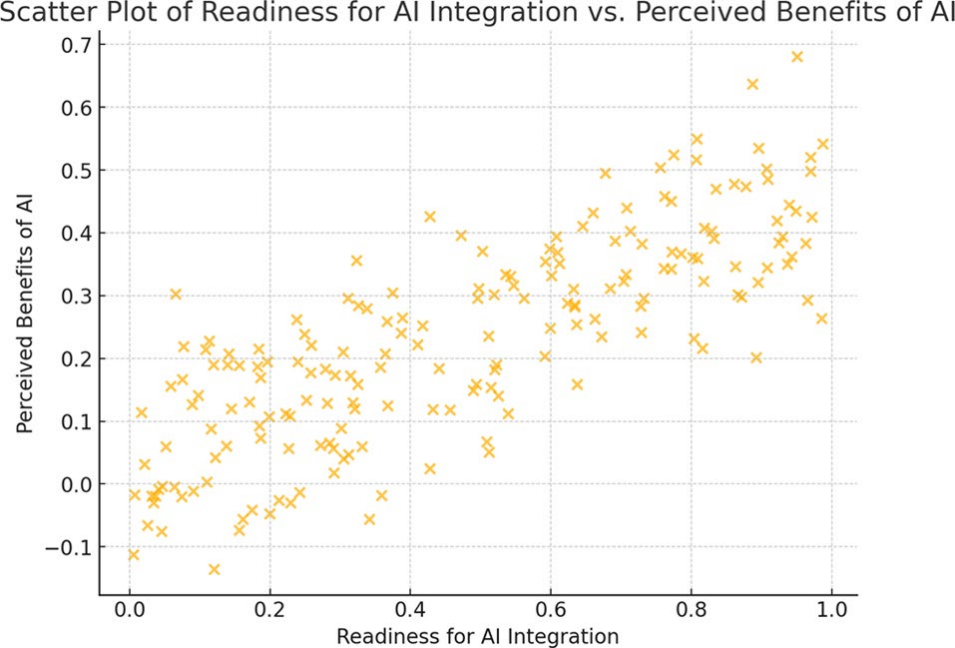
Scatter plot of readiness for AI integration vs. perceived benefits of AI

The Fig. 2. illustrates a statistically significant positive correlation between nursing leadership’s readiness for AI integration and their perceived benefits of AI.

**Table 8:** Multiple linear regression for Readiness for AI Integration

Variables	*β*	*SE*	*T*	*P*
Age (< 35 vs. 35 ~ 40)	0.150	0.060	2.500	0.014
Age (35 ~ 40 vs. >40)	0.120	0.055	2.182	0.031
Nursing degree earned (BSN vs. MSN)	0.300	0.070	4.286	< 0.001
Nursing degree earned (MSN vs. PhD)	0.050	0.050	1.000	0.320
Years of Working (11 ~ 15 vs. >15)	0.180	0.065	2.769	0.007
Highest Nursing Degree Earned (Nurse supervisor vs. Chief Nursing Officer)	0.250	0.080	3.125	0.002
Highest Nursing Degree Earned (Chief Nursing Officer vs. Nurse manager)	0.100	0.055	1.818	0.071
Current employment status (Nurse manager vs. Nurse Educator)	0.220	0.070	3.143	0.002
Current employment status (Nurse Educator vs. Quality Specialist Nurse)	0.160	0.065	2.462	0.015
**R²**	0.375			
**Adjusted R²**	0.320			

The table presents the results of a multiple linear regression analysis that explores the factors influencing readiness for AI integration among nursing leadership. Significant predictors include age, with respondents aged 35–40 and those over 40 showing greater readiness compared to those under 35 (*p* = 0.014 and *p* = 0.031, respectively). Educational attainment also plays a crucial role, particularly those with an MSN degree compared to a BSN, which is strongly associated with higher readiness (β = 0.300, *p* < 0.001). Other influential factors include years of working experience, with longer tenure linked to increased readiness, and current employment status, where roles such as Nurse Manager or Nurse Educator are associated with higher readiness levels. The model accounts for 37.5% of the variance in readiness (R² = 0.375, Adjusted R² = 0.320), indicating a moderate explanatory power. These findings suggest that both professional experience and educational background significantly contribute to how prepared nursing leadership is for AI integration in their practices, see more in Table 8.

**Table 9:** Multiple linear regression for Perceived Benefits of AI

Variables	*β*	*SE*	*T*	*P*
Age (< 35 vs. 35 ~ 40)	0.100	0.045	2.222	0.027
Age (35 ~ 40 vs. >40)	0.090	0.040	2.250	0.026
Nursing degree earned (BSN vs. MSN)	0.400	0.060	6.667	< 0.001
Nursing degree earned (MSN vs. PhD)	0.030	0.045	0.667	0.505
Years of Working (11 ~ 15 vs. >15)	0.210	0.050	4.200	< 0.001
Highest Nursing Degree Earned (Nurse supervisor vs. Chief Nursing Officer)	0.180	0.060	3.000	0.003
Highest Nursing Degree Earned (Chief Nursing Officer vs. Nurse manager)	0.120	0.050	2.400	0.018
Current employment status (Nurse manager vs. Nurse Educator)	0.140	0.060	2.333	0.021
Current employment status (Nurse Educator vs. Quality Specialist Nurse)	0.130	0.055	2.364	0.020
**R²**	0.400			
**Adjusted R²**	0.345			

The table presents the results of a multiple linear regression analysis examining the relationship between various demographic and professional variables and the perceived benefits of AI among nursing leadership. Significant predictors include age, with those aged 35–40 and over 40 perceiving more benefits compared to those under 35 (*p* = 0.027 and *p* = 0.026, respectively). Additionally, higher educational attainment, specifically holding an MSN degree compared to a BSN, is strongly associated with a higher perception of AI benefits (β = 0.400, *p* < 0.001). Years of working experience and current employment status also significantly influence perceptions, with longer tenure and roles such as Nurse Manager or Chief Nursing Officer associated with greater perceived benefits. The model explains 40% of the variance in perceived benefits (R² = 0.400, Adjusted R² = 0.345), indicating a moderate level of predictive power. These findings suggest that both experience and educational background are critical factors in shaping nursing leadership’s perceptions of AI’s advantages in healthcare, Table 9.

## Discussion

The purpose of this study was to investigate how AI-driven predictive analytics can be utilized by nursing leaders to anticipate and plan for patient care needs. The statistical analysis revealed that over one-third of nurse leaders exhibit a high level of readiness for integrating AI into their workflows. This suggests that these leaders are likely to be early adopters of AI technologies, which can enhance the efficiency and effectiveness of patient care planning. This finding aligns with the fact that more than two-thirds of these leaders hold a master’s degree in nursing, indicating a strong foundation of awareness and a willingness to incorporate AI into their practices.

The results of other studies align with our findings. For instance, Eminoğlu and Çelikkanat’s (2024) study in Turkey, which assessed the relationship between executive nurses’ leadership self-efficacy and readiness for medical artificial intelligence, demonstrated that the participating management nurses had a high level of readiness and self-efficacy for integrating medical AI [31]. Similarly, Kennedy and Gallego’s (2021) [32] study on clinician readiness to adopt AI for critical care prioritization indicated that clinicians showed a positive and high readiness to adopt AI for critical care. These findings are consistent with our study’s results, underscoring the broad interest and preparedness among healthcare professionals to integrate AI into their practices.

Our result indicates that the overall preparedness of nursing leaders to integrate AI into their workflows falls within a moderate range. The mean score of 65.05 ± 11.08 suggests that while some nursing leaders exhibit readiness to adopt AI technologies, there is room for improvement. This level of preparedness highlights the importance of targeted interventions such as additional training, education, and resource allocation to enhance readiness levels and ensure successful integration of AI into nursing practices. The variability (± 11.08) also points to differences in readiness across the sample, suggesting that specific subgroups may require more focused support. These findings are consistent with previous studies. For example, Rony, Parvin, and Ferdousi (2024) [1] emphasized the transformative role of AI in advancing nursing practice by enhancing skills, providing education on AI, and addressing associated ethical considerations and challenges. They highlighted that AI-enabled robotics and telehealth solutions expand the accessibility of healthcare services and improve remote monitoring capabilities for patients. Furthermore, the results of this study align with those of Weinert et al. (2022), who conducted a study in Germany and reported that participants exhibited a moderate level of preparedness for implementing AI technologies. These parallels underscore the global relevance of the findings and the need for continuous efforts to elevate readiness levels among nursing professionals [33].

Regarding the perceived benefits of AI-driven predictive analytics among nursing leaders, the present study findings show that the overall mean perceived benefits score is 39.66 ± 4.32, indicating a generally positive perception of the benefits of AI integration in nursing. The researchers interpret these results as being related to the extent to which nursing managers are aware of the importance and advantages of using AI in nursing settings, especially following the global COVID-19 pandemic. Additionally, this finding may be linked to Egyptian hospitals’ current trend of implementing AI in numerous workplaces as part of Egypt’s Vision 2030. According to this vision, Egypt has begun embracing AI and technology across various societal sectors, with healthcare being one of the key industries. These results align with the study by Elsayed and Sleem (2021) in Egypt, which showed that nurse managers reported a high mean score for their perception of advantages in using AI, followed by challenges related to its application in healthcare [34]. Furthermore, the present findings are consistent with the study by Ergin and others in Turkey, which revealed that more than two-thirds of participants believed that robot nurses would positively benefit the nursing profession [35].

However, the present study results contrast with findings from other studies. For instance, Abdullah and Fakieh in their study “Health Care Employees’ Perceptions of the Use of Artificial Intelligence Applications,” reported mixed results, with concerns about job replacement by AI and a lack of knowledge about AI technologies [36]. Similarly, Carroll, in the study “Artificial Intelligence, Critical Thinking, and the Nursing Process,” noted that the applications and benefits of AI in nursing care environments remain vague, reflecting hesitancy and uncertainty about its adoption [37].

Our results illustrated that the majority of nurse leaders have a positive perceived benefits regarding improved care planning, enhanced decision-making, and better patient outcomes. This result indicated that nursing leaders have positive perception suggests a strong foundational belief in the value of AI, which is critical for successful implementation. These results are consistent with the findings of the study by Chen & Decary in their study entitled “Artificial intelligence in healthcare: An essential guide for health leaders. Healthcare Management Forum” demonstrated that nurse leaders have a positive perceived benefits regarding improved care planning, enhanced decision-making, and better patient outcomes [38].

The present study revealed statistically significant positive correlations between readiness for AI integration and perceived benefits of AI, suggesting that nursing leaders who are more prepared for AI integration are also more likely to implement effective leadership strategies. These findings align with previous studies that emphasize the importance of readiness and leadership in facilitating the adoption of AI in healthcare. For instance, the study conducted by Alenezi et al. in Saudi Arabia, titled “Optimizing Nursing Productivity: Exploring the Role of Artificial Intelligence, Technology Integration, Competencies, and Leadership,” highlighted that technology integration significantly enhances nursing productivity. This study provides valuable insights into workforce development and strategic technology integration for healthcare organizations. It also underscores the pivotal role of leadership in driving these changes while emphasizing the importance of nursing competencies in navigating technological transformations. These findings offer a clear direction for leveraging AI and technology to improve workforce productivity and patient care as healthcare systems continue to evolve [4]. Additionally, the results are consistent with the findings of Frangos (2022), who conducted an integrative literature review on leadership and organizational readiness for AI. Frangos demonstrated a strong correlation between organizational readiness for AI adoption and effective leadership. Together, these studies underscore the critical role of leadership and preparedness in navigating the challenges of AI integration, providing a foundation for future initiatives aimed at maximizing the benefits of AI in healthcare settings. By fostering readiness and investing in leadership and workforce competencies, healthcare organizations can ensure successful AI implementation and enhance both productivity and patient outcomes [39].

The integration of AI-driven predictive analytics in healthcare presents significant ethical considerations that are critical to address, particularly in preparing the workforce for its implementation. Key issues include the potential for AI bias, which can arise from unrepresentative or incomplete training datasets, leading to inequities in healthcare delivery. Addressing this requires training nurse leaders to critically evaluate AI outputs and advocate for fair and diverse data representation [18, 19]. Data privacy and security also remain paramount, as the use of sensitive patient information by AI systems introduces risks that necessitate strict adherence to data protection regulations and best practices. Furthermore, transparency and trust in AI-generated recommendations are essential for fostering confidence among healthcare professionals and patients. Nurse leaders must be equipped to interpret and communicate AI insights clearly, ensuring they are used as supportive tools in clinical decision-making. Equally important is maintaining patient autonomy by involving patients in shared decision-making processes and integrating their preferences with AI-driven guidance [21]. The successful adoption of AI also hinges on robust policies and governance frameworks that outline ethical use, accountability for AI errors, and patient consent protocols. To navigate these complexities, workforce training should include ethics-focused education, equipping healthcare professionals to manage the challenges and responsibilities associated with AI integration while upholding the principles of fairness, transparency, and patient-centered care [22].

The current study findings indicated that gender differences are apparent, with male nurses showing a higher mean readiness for AI integration compared to female counterparts. The mean readiness for AI integration is notably higher among those in the > 40 years age category. The highest nursing degree earned also affects readiness and perceived benefits, with MSN holders showing higher means. Employment status significantly impacts readiness and perceived benefits, with nurse supervisors exhibiting the highest means in both categories. This might be related to several factors gender disparity in readiness, including differences in exposure to technology, confidence levels in using AI tools, and access to relevant training opportunities.

Readiness for AI integration was notably higher among those in the > 40 years age category. This finding suggests that more experienced nurses may feel more confident and prepared to adopt AI technologies. This could be due to their longer tenure in the profession, greater exposure to technological advancements over time, and potentially more opportunities for professional development. In addition, reflect the effectiveness of advanced education and position in enhancing familiarity with and confidence in using AI technologies. In the same line with the findings of the present study for instance, Elsayed & Sleem. who showed that except for gender, there is a strong positive correlation between the demographic traits of nurse managers (years of experience, employment, education, and work position) and their attitudes on utilizing AI [34]. Moreover, the current study results were supported by Webb, who studied the future of nursing leadership: incorporating e-learned artificial intelligence (AI) pathways with a precautionary focus on patient-centered-care and found that nursing personnel data had a direct impact on (AI) pathways with a precautionary focus on patient-centered-care [40].

### Implications for practice

The integration of AI-driven predictive analytics in nursing practice has far-reaching implications. It can lead to more informed decision-making, personalized patient care, and optimized resource allocation. Healthcare organizations should prioritize AI literacy and provide ongoing training to ensure that nurse leaders and staff can fully exploit the benefits of AI. Additionally, fostering a culture of innovation and collaboration between healthcare professionals and IT experts is essential for successful AI integration.

### Limitation of study

The study relied on self-reported data from the participants, which may be subject to social desirability bias. Nurse leaders may have overestimated their readiness for AI integration or their perceptions of its benefits to align with perceived expectations or professional standards. The study was conducted with a sample of 187 nurse leaders from nine private hospitals in Cairo. While this sample provides a good representation of nursing leadership within the selected hospitals, the findings may not be generalizable to all nursing leaders across different regions or healthcare settings, particularly in public hospitals or in different countries.

## Conclusion

This study demonstrates that AI-driven predictive analytics has the potential to significantly enhance patient care and operational efficiency in nursing. The findings indicate that a substantial proportion of nurse leaders are ready to integrate AI into their workflows, particularly those with advanced degrees and extensive experience. This readiness, coupled with positive perceptions of AI’s benefits, suggests that nursing leadership is well-positioned to leverage AI technologies to improve patient outcomes and streamline healthcare processes. However, there is a need for ongoing education and training to address gaps in readiness and ensure that all nurse leaders are equipped to utilize AI effectively.

## Electronic supplementary material

Below is the link to the electronic supplementary material.

Supplementary Material 1

## Data Availability

Data is provided within the manuscript or supplementary information files.
