# Supplementary material for: Empowering nurse leaders: readiness for AI integration and the perceived benefits of predictive analytics
Source: BMC Nurs. 2025 Jan 16;24:56. doi: 10.1186/s12912-024-02653-x (PMC11737245; doi:10.1186/s12912-024-02653-x)
Supplement: Supplementary file 1 — Supplementary Material 1 [file 12912_2024_2653_MOESM1_ESM.docx]

**Empowering Nurse Leaders: Readiness for AI Integration and the Perceived Benefits of Predictive Analytics**

**Characteristics of nurses:**

**Gender**

Male

Female

**Age**

Less than 35 years

35-40 years

> 40 years

**Highest Nursing Degree Earned**

Bachelor of Science in Nursing (BSN)

Master of Science in Nursing (MSN)

Nursing PhD.

**Current Employment Status**

Nurse supervisor

Chief Nursing Officer

Nurse manager

Nurse Educator

Healthcare quality specialist nurse

**Nursing Leaders' Readiness for AI Integration**

| **Item Description** | **Strongly disagree** | **Disagree** | **Agree** | **Strongly agree** |
| --- | --- | --- | --- | --- |
| Leadership is proactive in supporting AI implementation |  |  |  |  |
| Leadership encourages innovation related to AI |  |  |  |  |
| Leadership sets clear AI goals for the organization |  |  |  |  |
| AI implementation is a strategic priority in our organization |  |  |  |  |
| Leadership communicates AI benefits to staff |  |  |  |  |
| Staff members are open to learning about AI |  |  |  |  |
| Staff are enthusiastic about new AI technologies |  |  |  |  |
| Staff are confident in using AI in day-to-day tasks |  |  |  |  |
| Staff collaborate effectively when implementing AI solutions |  |  |  |  |
| Staff are regularly trained in AI technologies |  |  |  |  |
| We have the necessary technical infrastructure for AI |  |  |  |  |
| Our systems are equipped to support AI integration |  |  |  |  |
| AI technology is effectively integrated into our existing workflows |  |  |  |  |
| We update our AI technology regularly |  |  |  |  |
| We have access to the resources needed for successful AI implementation |  |  |  |  |
| Our AI systems are secure and protect patient data |  |  |  |  |
| We use data-driven insights from AI to inform decision-making |  |  |  |  |
| Leadership encourages AI training and development for staff |  |  |  |  |
| AI helps us improve patient outcomes |  |  |  |  |
| AI initiatives are aligned with our organization's overall mission |  |  |  |  |

**Perceived Benefits of AI-Driven Predictive Analytics**

| **Item Description** | **Strongly disagree** | **Disagree** | **Neutral** | **Agree** | **Strongly agree** |
| --- | --- | --- | --- | --- | --- |
| AI enhances patient care by providing real-time predictive insights |  |  |  |  |  |
| AI helps in anticipating patient needs and improving outcomes |  |  |  |  |  |
| AI-driven analytics contributes to reducing patient complications |  |  |  |  |  |
| AI assists in optimizing patient treatment plans |  |  |  |  |  |
| AI helps identify potential risks before they become critical |  |  |  |  |  |
| AI enhances the decision-making process by providing data-driven recommendations |  |  |  |  |  |
| AI reduces uncertainty in decision-making during patient care planning |  |  |  |  |  |
| AI allows for faster and more accurate decision-making |  |  |  |  |  |
| AI tools integrate well with existing decision-support systems |  |  |  |  |  |
| AI helps to personalize patient care based on predictive data |  |  |  |  |  |
